# Pairwise graph edit distance characterizes the impact of the construction method on pangenome graphs

**DOI:** 10.1093/bioinformatics/btaf291

**Published:** 2025-05-09

**Authors:** Siegfried Dubois, Matthias Zytnicki, Claire Lemaitre, Thomas Faraut

**Affiliations:** Univ Rennes, CNRS, Inria, IRISA-UMR 6074, Rennes F-35000, France; GenPhySE, Université de Toulouse, INRAE, ENVT, Castanet-Tolosan 31320, France; MIAT, INRAE, Castanet-Tolosan 31320, France; Univ Rennes, CNRS, Inria, IRISA-UMR 6074, Rennes F-35000, France; GenPhySE, Université de Toulouse, INRAE, ENVT, Castanet-Tolosan 31320, France

## Abstract

**Motivation:**

Pangenome variation graphs are an increasingly used tool to perform genome analysis, aiming to replace a linear reference in a wide variety of genomic analyses. The construction of a variation graph from a collection of chromosome-size genome sequences is a difficult task that is generally addressed using a number of heuristics. The question that arises is to what extent the construction method influences the resulting graph, and the characterization of variability.

**Results:**

We aim to characterize the differences between variation graphs derived from the same set of genomes with a metric which expresses and pinpoint differences. We designed a pairwise variation graph comparison algorithm, which establishes an edit distance between variation graphs, threading the genomes through both graphs. We applied our method to pangenome graphs built from yeast and human chromosome collections, and demonstrate that our method effectively characterizes discordances between pangenome graph construction methods and scales to real datasets.

**Availability and implementation:**

*pancat compare* is published as free Rust software under the AGPL3.0 open source license. Source code and documentation are available at https://github.com/dubssieg/rs-pancat-compare. Snapshot available on Software Heritage at swh:1:dir:61acda8ba3dac1709ed60530147d3871831be629.

## 1 Introduction

One of the primary aims of genetics is to analyze how genetic variability impacts phenotype variability. In the standard approaches of genomic analysis, we use the concept of reference sequence: a fully resolved, high quality assembly which stands for a golden standard for a species.

However, a single sequence cannot encapsulate the whole diversity of a population; nested variations and complex structural variants cannot be positioned over a reference genome and represented as a table with confidence (Paten *et al.* 2017). Rather than considering each of these genomes individually, the aim of the pangenomic approach is to combine the information from multiple genomes. It is the core of the pangenomic approach, which aims to aggregate sequence information from multiple genomes, improving the quality of read mapping and variant genotyping (Abel *et al.* 2020, Sirén *et al.* 2021). To aggregate this information, a data structure, the variation graph, has been introduced. It is a sequence graph where each genome is embedded as a path in the graph with the consecutive nodes corresponding to successive segments on the associated genome sequence (Baaijens *et al.* 2022). By its construction, variations emerge from the graph topology. In such structures, shared sub-paths correspond to shared genomic regions between genomes and divergent paths to variations, whether small or large, such as inversions, insertions, deletions, and substitutions.

The faithful representation of variability is a key to any downstream analysis. Variant genotyping using variation graphs relies heavily on the structural analysis of the graph, including features like bubbles (Hickey *et al.* 2020). To ensure accurate reporting of variants, the graph must faithfully represent the variations through the arrangement of nodes. If this is not achieved, variants may be reported inaccurately or not at all. Additionally, a poorly constructed graph can induce errors, creating false variants. A significant portion of the quality is derived from the genomes used, but the choices made by the algorithms also substantially impact the final results. Construction methods for these structures are relatively recent, with *state-of-the-art* pangenome builders such as Minigraph-Cactus (Hickey *et al.* 2023) and PGGB (Garrison and Guarracino 2023) receiving frequent updates. Inducing a variation graph from a collection of chromosome-size genome sequences poses a significant challenge that is generally addressed through various heuristics, starting with the choice of an alignment algorithm. Moreover, as there is no exact description of the ideal properties for a variation graph (Cicherski and Dojer 2023), various approaches for criteria to optimize have been developed.

In the current literature, variation graphs are described mainly with simple metrics such as the number of nodes and edges. Pangenome graph builders use different alignment algorithms, do not have the same rules for graph induction and apply different post-processing steps. Recent studies have shown that using multiple tools on the same input data produces different graphs (Leonard *et al.* 2022, Andreace *et al.* 2023, Liao *et al.* 2023); however, there is no method to quantify or qualify the differences between them. Recently, a method to compare the contents of two graphs using elastic-degenerate strings (Zuba *et al.* 2024) was published, which addresses the question of the difference of sequence content between graphs, but not how the same sequences are differently embedded in the graph.

Pairwise graph comparison is a well-studied topic, with various measures and algorithms (Wills and Meyer 2020) for general purpose graphs that rely vastly on topology. Variation graphs are particular graphs that are made of labeled nodes representing parts of the genomes. Those labels and the way they are scattered in the graph structure convey meaningful information about the potential conservation or variation of sequences, which must be considered when comparing graphs.

In the work presented here, we aim to characterize the differences between variation graphs derived from the same set of genomes. To pursue this objective, we present an algorithm to compute a pairwise segmentation distance between variation graphs, exploiting the way the genomes are embedded in the graph structure. Our method not only provides a metric to evaluate the extent to which two graphs differ but also the ability to pinpoint where differences are, allowing to analyze their distribution within the graph and to locate them on the nodes. Then, we apply this method to real pangenome graphs to compare graphs built from two state-of-the-art pipelines, and assess their differences. Lastly, we investigate these differences—their impact, position, and size—in order to provide insights into their impact on pangenome analysis.

## 2 Materials and methods

### 2.1 Sequence graphs and genome segmentation

Let $\Gamma =\{\Gamma_{0},\Gamma_{1},\ldots \Gamma_{n}\}$ be a set of genomes. A genome $\Gamma_{i}$ is represented by a string $w_{1}\ldots w_{m}$ with *m* being the size of the genome, on the alphabet {A,C,G,T}.

This genome collection Γ can be represented by a sequence graph G(Γ)=(V,E). This structure is a directed graph where each node v∈V is labeled by a word *w*, that exists in at least one genome of Γ, in forward or reverse orientation. Any edge e∈E links two vertices whose labels are contiguous in at least one genome. Edges are directed and annotated at both ends, conveying both reading direction and order of connected nodes.

A pangenome graph G(Γ)=(V,E,P) is a sequence graph extended by a collection of paths P. A path consists of an oriented and ordered list of vertices linked by edges in the graph. A path represents a continuous input sequence, be it a scaffold or a chromosome; meaning a single genome can be embedded in the graph by numerous paths; for the sake of simplicity, we will consider that each genome is represented as a single path although our method can be generalized to encompass graphs with genomes split into several chromosomes or scaffolds. We require the pangenome graphs to be *complete*. We say that a pangenome graph is *complete* if the sequence of each element of Γ can be read by following one path from the path collection P (Definition 1).

Definition 1 (Complete pangenome graph).

A complete pangenome graph G(Γ)=(V,E,P) is a pangenome graph where |P|=|Γ|, and every genome of Γ is represented in exactly one path of P. Concatenation of every label associated with the vertices of $P_{i}$, in respect with their orientation, is exactly the sequence of $\Gamma_{i}$.

By the definition of a pangenome graph, the successive nodes encountered in the traversal of path $P_{i}$ correspond to consecutive genomic intervals along the genome $\Gamma_{i}$ associated to this path. We can consider a path as an ordered and oriented list of contiguous genomic intervals on a genome. This describes a *segmentation* of each genome in the graph, where genomic intervals defined by the traversed vertices are separated by *breakpoints*.

Definition 2 (Breakpoint).

A breakpoint b is a position in a genome where the graph structure breaks the continuity between two consecutive genomic intervals. The existence of a breakpoint on a genome $\Gamma_{i}$ is associated with an edge between the two vertices supporting the two associated labels in the path $P_{i}$. This position is expressed in number of basepairs from the start of the genome $\Gamma_{i}$.

We define $B_{i}=b_{0}\ldots b_{n}$ as the set of breakpoints on $\Gamma_{i}$ induced by G. This set of breakpoints corresponds to a segmentation of $\Gamma_{i}$, which reflects the evolutionary relationship with the other genomes in Γ but also heavily depends on the graph construction process.

Our approach seeks to identify differences between two variation graphs. It is to be tailored to the specific types of differences we aim to detect. When comparing two variation graphs, one important question is how they differ in the genetic variability they encode. In a graph, the breakpoints define the limits of genomic variants. Differences in breakpoint positions for a given genome therefore imply that the genomic variants involving this genome are defined or represented differently (see some examples in Supplementary Fig. S1). In order to compare the graphs, we propose to compare how differently the same genomes are segmented in the two graphs, resulting in comparing pairwisely breakpoint sets for each genome.

We propose in the following paragraphs an algorithm which compares two pangenome graphs on the basis of the pairwise comparison of breakpoint sets associated to each genome of Γ. Let $G_{a}(\Gamma )=(V^{a},E^{a},P^{a})$ and $G_{b}(\Gamma )=(V^{b},E^{b},P^{b})$ be two complete pangenome graphs sharing the same set of genomes Γ. The idea of our comparison method is to find differences between the two segmentations of $\Gamma_{i}$ by uncovering the smallest set of breakpoints that differentiates $P_{i}^{a}$ and $P_{i}^{b}$.

We define two reciprocal operations, *merge* and *split*, which correspond respectively to the suppression and addition of a breakpoint in a path at a position *x*. Thus, $\mathrm{merge}(x,P_{i}^{a})$ removes the breakpoint at the position *x* in the path $P_{i}^{a}$, while $\mathrm{split}(x,P_{i}^{b})$ adds on the path $P_{i}^{b}$ a breakpoint at the position *x*. Merges and splits are particular breakpoints that are missing in $B_{i}^{a}$ or in $B_{i}^{b}$. If we consider $B_{i}^{a}$ and $B_{i}^{b}$ as two sets, the symmetric difference of the breakpoints of the two segmentations is the union of all required merges and splits, and we call the size of this set the segmentation distance (Definition 3). Summation of all segmentation distances between two graphs gives the distance between them (Definition 4).

Definition 3 (Path segmentation distance).

The segmentation distance $d_{s}$ for a genome $\Gamma_{i}$ present in two graphs $G_{a}$ and $G_{b}$ is the minimum set of operations enabling transforming one segmentation into the other. It corresponds to the breakpoints that are exclusive to one of the breakpoints sets $B_{i}^{a}$ and $B_{i}^{b}$.

$$d_{s}(P_{i}^{a},P_{i}^{b})=|B_{i}^{a}⊖B_{i}^{b}|$$

Where x⊖y is the symmetric difference between x and y.

Definition 4 (Graph segmentation distance).

The segmentation distance d between two graphs containing the same genome set Γ is the summation of all the segmentation distances over the genomes of Γ:

$$d(G_{a},G_{b})=\sum_{i=1}^{\left|\Gamma \right|}d_{s}(P_{i}^{a},P_{i}^{b})$$

We can generalize this definition to any pair of graphs that shares at least one common genome, by computing the intersection of paths of the two graphs and applying the segmentation distance computation only on the intersection.

### 2.2 Algorithm

For each genome $\Gamma_{i}$, we browse linearly and simultaneously through the two associated segmentations to find the specific breakpoints between the two breakpoint lists. In Algorithm 1, $B_{\alpha }^{a}=x_{0},x_{1}\ldots x_{n}$ and $B_{\alpha }^{b}=y_{0},y_{1}\ldots y_{m}$ are breakpoints of two paths $P_{\alpha }^{a}$ and $P_{\alpha }^{b}$ representing $\Gamma_{\alpha }$, such that both paths represent the same sequence but can differ in segmentation. We consider at each step a position *p* in the string $\Gamma_{i}$, which increases at each iteration to the next closest breakpoint across both segmentations. M is the set storing merges, and S the set storing splits.

Algorithm 1. Distance $d_{s}$ between two segmentations of $\Gamma_{\alpha }$

1: **Input**  $B_{\alpha }^{a}$, $B_{\alpha }^{b}$, $\Gamma_{\alpha }$

2: **Init**  M←∅, S←∅

3: **Init**  $p\leftarrow \min(B_{\alpha }^{a}[0],B_{\alpha }^{b}[0])$

4: **Init**  i←0, j←0

5: **while**  $p<|\Gamma_{\alpha }|$  **do**

6:    **if**  $p=B_{\alpha }^{a}[i]$ and $p=B_{\alpha }^{b}[j]$  **then**

7:     i←i+1

8:     j←j+1

9:        **else if**  $p=B_{\alpha }^{a}[i]$  **then**

10:     i←i+1

11:     M←M∪{p}

12:     **else**

13:     j←j+1

14:     S←S∪{p}

15:     **end if**

16:     $p\leftarrow \min(B_{\alpha }^{a}[i],B_{\alpha }^{b}[j])$

17: **end while**

18: **return**  |M∪S|

Each of our operations applies on a single breakpoint, by adding it or removing it in the other segmentation. The number of differing breakpoints across both segmentations is the summation of the breakpoints that are specific to any of the two segmentations: $B^{a}⊖B^{b}$. When we iterate, we take into account every breakpoint ($B^{a}\cup B^{b}$) but we only count as edit breakpoints that are not common to both segmentations ($B^{a}\cup B^{b}\setminus B^{a}\cap B^{b}$), which is the symmetric difference between the two sets, thus ensuring that our algorithm gets the minimal number of edits. As the symmetric difference between the sets is a XOR operation which has been proved to be a distance (Maymounkov and Mazières 2002), we can say that Algorithm 1 finds the distance between two segmentations of a genome (Supplementary Text S1).

Algorithm 1 executes for a genome $\Gamma_{i}$ with two breakpoints sets $B_{i}^{a}$ and $B_{i}^{b}$ in a total of $m=|B_{i}^{a}\cup B_{i}^{b}|$ steps. It results in a complexity of O(m) for the path segmentation distance. When a segmentation distance between two graphs is computed, as we execute this algorithm once per genome, we end up with a complexity of O(n·m) with *n* being the number of genomes in Γ and *m* the size of the union of the breakpoints of the two segmentation of each genome. Note that the number of breakpoints per genome is likely to increase with the number of genomes, as the pangenome graphs get more complex. In the worst case, the number of breakpoints per genome is the size of the genome, therefore the worst case time complexity of this algorithm is linear with the total number of nucleotides in the genome collection.

### 2.3 Spurious breakpoints

In a pangenome graph, some breakpoints might be associated with nonbranching paths. They may result from the graph construction process, e.g. when long nodes are chopped in smaller parts for performance reasons. Contrary to genuine breakpoints, they do not model genomic variation and, ideally, should not be taken into account when computing the segmentation distance. We call them spurious breakpoints. Spurious breakpoints can be filtered out with our method before computing the distance, and masking them allows for a better representation of the real differences between graphs. In the general case, we recommend to mask them to negate the impact of node chopping on the graph.

### 2.4 Case study

The algorithm was applied both on published pangenome graphs, the HPRC human draft pangenome graphs (Liao *et al.* 2023), and graphs built from telomere-to-telomere yeast assemblies (O’Donnell *et al.* 2023).

For the yeast dataset, we selected chromosome 1 from 15 samples and constructed the graphs using the Minigraph-Cactus pipeline (Hickey *et al.* 2023) (MC, v2.9.0) and the PanGenome Graph Builder (Garrison *et al.* 2024) (PGGB, v0.6.0). From the selected yeast assemblies, we built multiple graphs, varying the reference sample for MC and the order of secondary genomes. We also constructed a corresponding PGGB graph with the same assemblies, ensuring that all comparisons were made between graphs representing the same input data exactly. All graphs were verified as complete pangenome graphs. Graphs built with MC had the *clip* and *filter* parameters set to zero, ensuring that the entire sequence was embedded in the structure. Both tools were run with all other parameters kept at their default settings. The graphs did not undergo any post-processing.

For the human dataset, we analyzed differences between MC and PGGB graphs from the HPRC dataset (year 1) for chromosomes 1 and 21 individually. We computed edits between the full MC CHM13 graph (built with MC v2.6.4, HPRC year 1, v1.1), without clipping or filtering, and the PGGB graph (built with PGGB v0.2.0 + 531f85f, HPRC year 1, v1.0).

Human pangenome graphs from the HPRC can be challenging to compare due to differences in scaffold sets. Not all genomes included in these graphs are telomere-to-telomere, thus implying that chromosomes may be composed of multiple scaffolds. The assignment of these scaffolds to chromosomes was performed using distinct methods for the two tools, resulting in graphs that include both shared sequences and tool-specific sequences (see Supplementary Table S1). In this case, the distance is computed on the intersection of scaffolds of the two graphs.

We identified genomic variants in yeast graphs using *vg deconstruct* (Hickey *et al.* 2020) (v1.56.0). For human graphs, variants were extracted from the raw CHM13 VCFs supplied along the pangenome graphs. In this study, for the purpose of comparing graphs, we make a distinction between “graph-shared variants” and “graph-private variants.” A “graph-shared variant” is defined as a variant that has the same position, reference allele, and alternative allele sequences in both graphs (ignoring the order of alternative alleles). In contrast, a “graph-private variant” refers to any variant that does not meet these criteria.

Tandem repeats on the linear reference genome were identified using *TandemRepeatFinder* (Benson 1999) (v4.09.1). Segmental duplications in the yeast genome were detected with *BISER* (Išerić *et al.* 2022) (v1.4.0) and retrieved from CHM13 annotations for human (Vollger *et al.* 2022). Centromeric, telomeric and subtelomeric regions were collected from genome annotations (Altemose *et al.* 2022, O’Donnell *et al.* 2023).

Yeast graphs and Supplementary Data are available at https://doi.org/10.5281/zenodo.10932489.

### 2.5 Implementation

All edits between graphs were computed using *rs-pancat-compare* (v0.1.4), which is our Rust implementation of Alg. 1 available at https://github.com/dubssieg/rs-pancat-compare. After storing a mapping of the nodes to their respective length, we do a parallel reading of the node lists described in the paths and asynchronously iterate on both paths to compute each operation. In every comparison, the --*spurious* flag was set.

The output is a tab-separated file containing one edit per line described by its path name, its position in the path, a one-letter encoding of the operation, nodes on which the operation applies to in first and second graphs, and individual ending breakpoints for both nodes. Results are subsequently processed using Jupyter Notebooks, which are available at https://github.com/dubssieg/pancat_paper.

## 3 Results

### 3.1 Comparing graphs

We applied our method to two datasets: yeast graphs built from 15 telomere-to-telomere assemblies of chromosome 1 (O’Donnell *et al.* 2023) and graphs of chromosome 1 and 21 from the HPRC human draft pangenome (Liao *et al.* 2023). We computed metrics for both the Minigraph-Cactus (MC) and the PanGenome Graph Builder (PGGB) versions of the graphs and measured the distances between them (Table 1).

**Table 1.** Datasets used to evaluate the comparison tool.^a^

Org.	Chr.	#hap.	Length (bp)	Tool	Total length (bp)	Nodes	Edges	Variants	Merges	Splits
Yeast	1	15	222 424	MC	386 083	34 889	48 130	9213	35 956	44 097
				PGGB	350 140	27 213	37 755	8 626		
Human	21	90	45 090 682	MC	423 514 455	2 541 744	3 486 748	574 574	12 949 063	79 480 033
				PGGB	273 835 317	2 760 531	3 882 969	841 419		
Human	1	90	248 387 328	MC	1 449 752 909	6 951 299	9 620 356	2 224 237	32 362 430	189 702 647
				PGGB	1 117 392 094	11 109 656	15 398 037	3 456 614		

a MiniGraph-Cactus = MC, PanGenome Graph Builder = PGGB. Org: Organism. Chr: chromosome number. #hap: haplotype count in graph. Length: length of the reference genome. Total length: sum of all node lengths in graph. Variants: Variants are called against the haplotype that was used as reference to build the MC graph. Merges and splits are operation counts computed in the direction from the MC graph to the PGGB one.

#### 3.1.1 Differences between pangenome graphs

For the yeast dataset, the graphs of the chromosome 1, constructed from the same 15 genome set with MC and PGGB show noticeable differences for every metric (Tab. 1). The PGGB graph is smaller in all aspects, containing 27 213 nodes (compared to 34 889 for MC) and a total length of 350 kb (compared to 386 kb for MC). The length of a graph is computed by summing the length of all the labels of its nodes. Representing the same genome content with a reduced length can result from a failure of one of the graph to summarize shared genome content or a tendency of the other graph to adopt a more compact representation, e.g. for the representation of repeated sequences, resulting in an increased number of cycles in the genome paths. This is a known difference between MC and PGGB, with the latter constructing more compact graphs at the expense of a larger number of cycles (Leonard *et al.* 2022). These differences are also reflected in the variant sets reported by *vg deconstruct*, with 9213 variants identified in the MC graph and 8224 in the PGGB graph. Among these variants, 6291 are shared between the two graphs, 2922 are unique to the MC graph, and 1933 are unique to the PGGB graph. For the same chromosome, analysis of the operations needed to transform the MC graph into the PGGB graph reveals that 44 907 splits and 35 956 merges are needed. By dividing by the total number of characters in the genome collection, these numbers represents an average of 25.24 edits per kilobase.

For the human dataset, the observed metrics follow the same trends. The density of edits also remains within a similar range: the chromosome 21 graph shows an average of 25.33 edits per kilobase, while chromosome 1 displays approximately 10.22 edits per kilobase.

It has to be noted that the two graphs do not share exactly the same set of scaffolds. For each HPRC graph (MC or PGGB), the scaffolds included in the pangenome graph for each chromosome is defined by the construction strategy used that apparently differed (see Supplementary Table S1). This might impact the segmentation of the other scaffolds in the graphs and possibly of the segmentation of the shared scaffolds which will be captured by our algorithm and identified as graph discrepancies. These scaffolds, however, represent a small fraction of the whole scaffold set (6.24% for the chromosome 21 and 1.84% for the chromosome 1). We believe therefore that this does not change the global picture and that the observed differences result essentially from the construction algorithms rather than from the difference in the initial set of scaffolds.

#### 3.1.2 Distribution of edits among the nodes

Edits act on the nodes and are defined at the nucleotide level. When running the algorithm, each operation (split or merge) can be viewed as a split simply by exchanging the roles of the two segmentations being compared. We can therefore characterize each operation with respect to the node being split. The localization of edits within the nodes reflects the nature of the discrepancies between the representation of variants. We therefore define the position of an edit as the distance, in nucleotides, from the nearest node border, which we will refer to as the edit position. A position near the border suggests a difference in variant breakpoint placement rather than the existence of noticeably different variants. We observe that the distribution of edit positions is skewed toward the borders of the nodes (Supplementary Fig. S2B).

Since multiple edits can occur on the same node, the edit position does not capture the complete picture. We define the edit size as the distance, in nucleotides, between the edit position and the nearest edit or node border. Each operation breaks a node in two segments and the edit size is simply the size of the smallest segment produced by this single operation. Most of the edits are small: one-sized edits represents 83.61% of the total number of edits for yeast chromosome 1 (mean size 1.49 bp), 90.60% for human chromosome 21 (mean size 1.35 bp), and 86.04% for human chromosome 1 (mean size 1.92 bp).

Most of the edits are affecting the smallest nodes. More than 90% of the edits are located on nodes with length ≤50 bp although those nodes cover no more than 40% of the graph size (see Supplementary Fig. S2A). However, some large nodes can exhibit a very large number of edits. A single node, e.g. of the yeast MC chromosome 1 graph has to be edited 2339 times to match the corresponding nodes in the PGGB graph. In a similar manner, the human MC chromosome 21 graph has a node that has 1 354 801 edits, and the human MC chromosome 1 graph has a node that has 3 660 206 edits. It seems that those are nodes that were left nonaligned by one tool but where the other one forced the alignment.

#### 3.1.3 Impact of the reference choice

**Figure 1.**
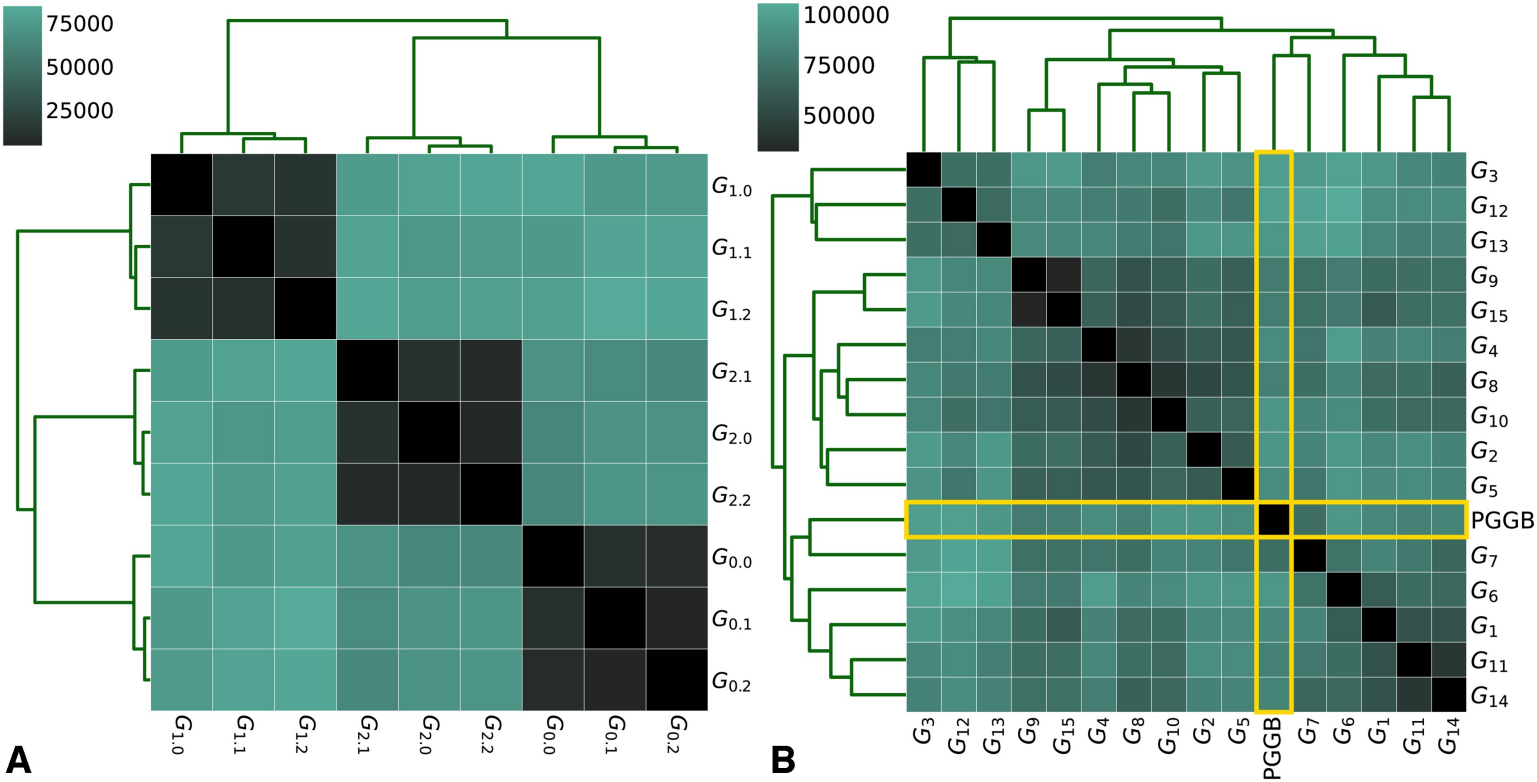
Hierarchical agglomerative clustering of distances between yeast graphs, built from the same set of genomes, computed with *pancat*. A lower distance means that less breakpoints needs to be added or removed to form the other graph. (A) Comparison of 9 MC graphs; each triad uses a shared reference and only differs by secondary genomes order. (B) Comparison of 15 MC graphs and 1 PGGB graph; MC graphs differs by the genome used as reference.

The MC pipeline adds genomes incrementally atop of a chosen reference. We applied our method to compute segmentation distances between graphs built with different reference genomes and input genome orders. According to the edit metric, in MC, changing the order of secondary genomes has less impact than switching the reference genome (Fig. 1A). Graphs that share the same reference genome cluster very well, with edits counts between 5k and 15k, whereas distances between graphs with different reference genomes range from 65k to 80k edits.

When comparing graphs made with MC featuring different references to the PGGB graph that does not take any reference nor genome order, it appears that changing the reference in MC can have more impact on segmentation distance than switching tools. Graphs generated with MC do not cluster separately from the PGGB graph with the same genomes. On average, the distance between the PGGB graph and MC graphs is 88k, compared to 78k between MC graphs. However, for almost every MC graph we built, there exists another MC graph that has a higher distance to the first than to the PGGB graph. This confirms the distance clustering (Fig. 1B), as the PGGB graph is not an outlier in terms of distance from the multiple MC graphs.

This observation has significant implications for graph analysis, as the differences directly affect the number of graph-shared variants and in turn the number of graph-private variants observed between graphs. The number of graph-private variants—those that differ in their detection or representation across graphs—is correlated with the number of edits (Spearman *r* = 0.82, *P*-value = 4.43e−64) whereas it is not the case for graph-shared variants (Spearman *r* = −0.07, *P*-value = 3.00e−01). This strong correlation with graph-private variants supports the idea that our segmentation distance serves as a robust metric for identifying areas featuring differently reported variants (see Supplementary Fig. S3).

### 3.2 Distribution along the genome

The segmentation distance not only provides a global measure of differences between two graphs but can also be decomposed locally into the edit sequence impacting the segmentation along a given genome. We will show that these edits are not distributed evenly.

#### 3.2.1 Edit hotspots

**Figure 2.**
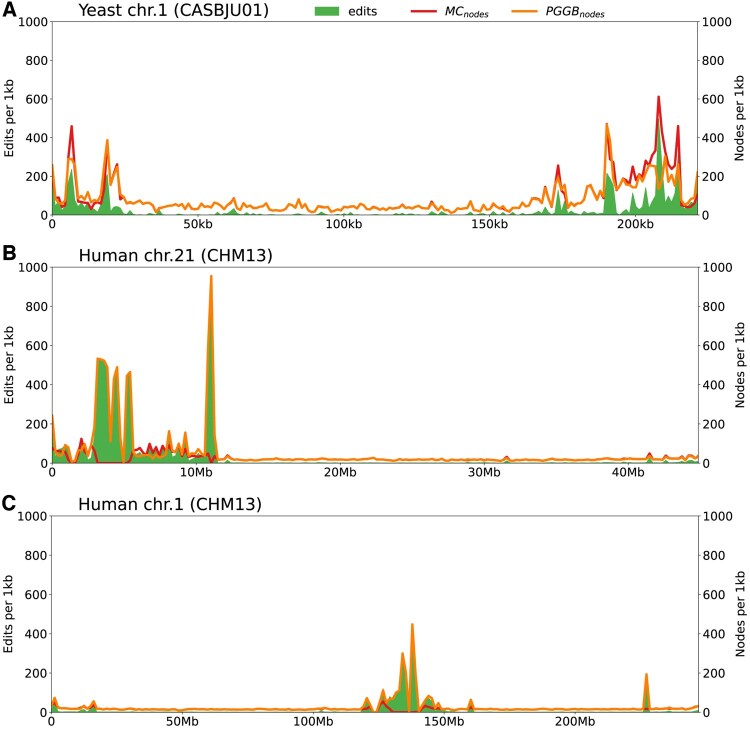
Visualization of edits and node counts along a selected linear genome. Only edits affecting this specific path in the graph are displayed, along with the corresponding node counts in both graphs. For each panel, the selected linear genome is the one that served as the reference in the MC graph. Genomes are segmented into windows, with edits and node counts expressed in units per kilobase.

Plotting the density of edits impacting a given genome in sliding windows along this same genome reveals that edits are not evenly distributed. We observe concentrations in some regions, creating hotspots of differences (Fig. 2). This behavior is consistent across all datasets. The same pattern, an uneven distribution of edits along the chromosomes with concentrations in subtelomeric regions and in centromeric regions, is observed when changing the reference for graph construction, as well as when projecting onto other haplotypes within the same graph.

#### 3.2.2 Relationship with the representation of variants

**Figure 3.**
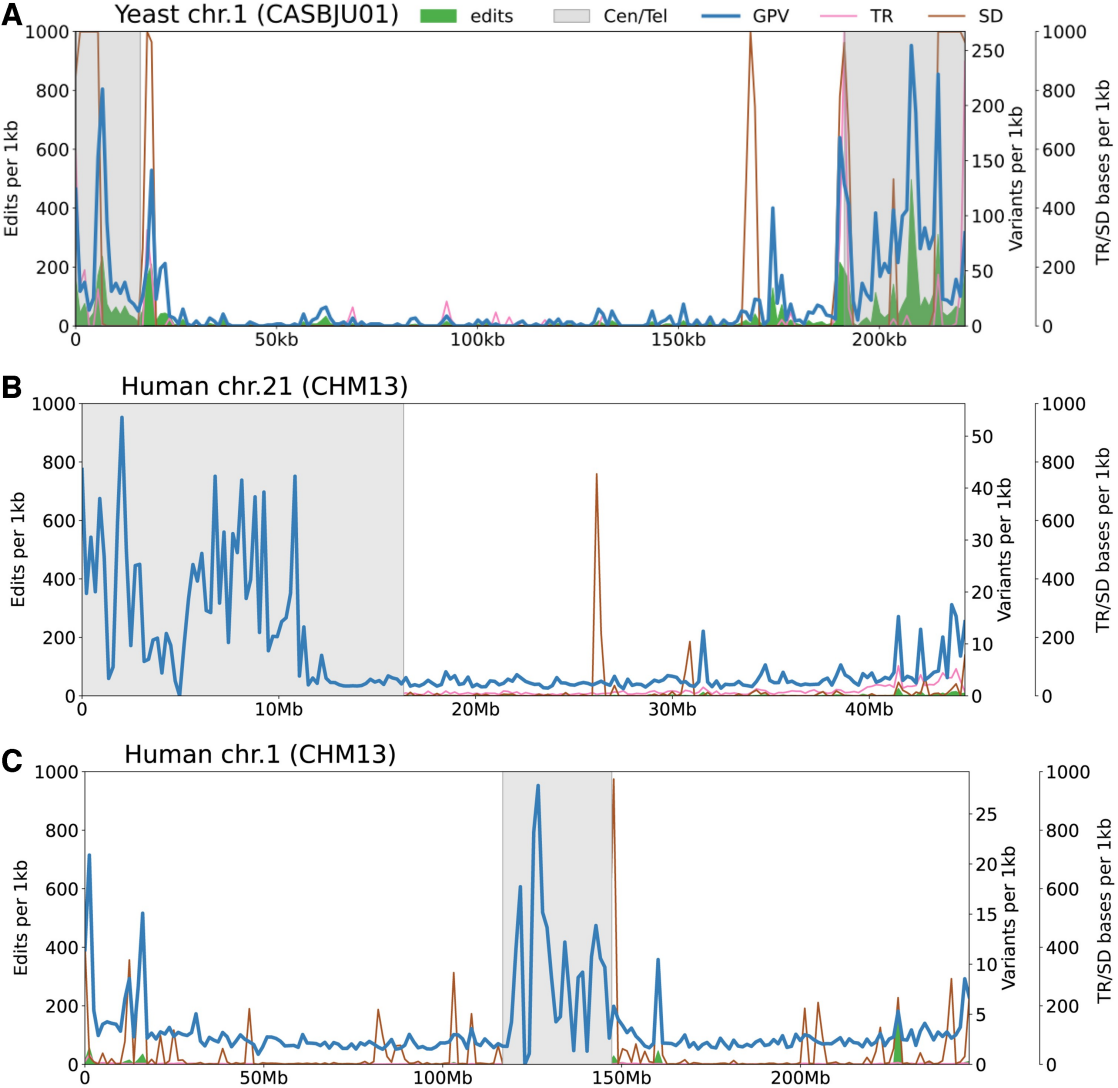
Visualization of edits, graph-private variants (GPV), tandem repeats (TR), and segmental duplications (SD) along a selected linear genome (the reference genome in MC graph). Tandem repeats and segmental duplications are expressed as a number of bases covered by these features per kilobase, and graph-private variants as a number of variants per kilobase. Grey boxes (Cen/Tel) represents annotated telomeric and subtelomeric regions for yeast (A), telomeric and centromeric annotations for human (B and C).

The differences between graphs should be reflected in differences in the representation of variants within the graphs. To confirm this, we extracted the variations between the genomes and a genome taken as reference (CASBJU01 for yeast and CHM13 for humans) using vg deconstruct. We distinguished between two types of variants: shared variants, which are observed in both graphs, and private variants, which are found in only one of the two graphs and which we will refer to as graph-private variants (see Section 2). For yeast chromosome 1, 56.44% of reported variants are shared, 26.22% are graph-private to MC, and 17.34% to PGGB. The comparison of the distribution, along the reference genome, of graph-private variants relative to this genome and the distribution of edits impacting this genome confirms a strong correlation (Fig. 3 and Supplementary Figs S4 and S5).

#### 3.2.3 Relationship between genome composition and graph

The regions with high concentrations of edits correspond to the subtelomeric regions in yeast which are known to be hypervariable regions (Yue *et al.* 2017). In human, these are centromeric regions and, to a lesser extent, regions near the telomeres (subtelomeric regions) (Fig. 3). These regions are also known to be hypervariable regions between individuals (Kim *et al.* 2025), and to harbor specific sequences, such as repeated sequences. Such sequences present alignment challenges which in turn have an impact on the graph construction process. To investigate this, we plotted the distribution, along the genome, of segmental duplications (SD) and tandem repeats (TR) superimposed on the distribution of edits (Fig. 3). While some edit peaks correspond with high density SD or TR regions, some of the high concentrations of edits do not seem to be associated with these features (see also the scatterplots and correlation values in Supplementary Figs S4 and S5). The strong association between these edit-rich regions and high variability between genomes highlights the difficulty of characterizing genomic variability in these regions using existing methods.

### 3.3 Scalability

Our implementation scales reasonably well, with the comparison of graphs of human chromosomes with 90 haplotypes from the HPRC ranging from 5 min to 18 min (respectively for chromosome 21 and 1) using at most 1.2 GB for the human chromosome 1 (Table 2). We compared the entire human pangenome built with MC and PGGB with 20.3 Gb of RAM in 23 min and 18 s. The running time is related to the length of the paths, in nodes, and RAM growth is conditioned by the number of nodes in both graphs.

**Table 2.** Comparison of time and peak memory over diverse datasets.^a^

Organism	Chromosome	Wall time	RAM_*peak*_
Yeast	1	0 min 01 s	3.0 MB
Human	21	5 min 08 s	383 MB
	1	17 min 42 s	1.2 GB
	All	23 min 18 s	20.3 GB

a Peak memory takes into account the overhead of loading graphs in memory. When comparing all human graphs, each MC chromosome graph was compared to its analogous graph from PGGB (chromosomes 1 to 22 plus M, X, and Y). Timings and memory consumption were recorded using *heaptrack*. Results are collected from jobs executed on a laptop equipped with a 10-core 13th Gen Intel^®^ Core™ i7-1365U @ 3.6GHz (with hyper-threading and Turbo Boost on). Detailed system specifications are available at https://github.com/dubssieg/pancat_paper.

## 4 Discussion

In this work, we presented a novel method to compute a distance between a pair of variation graphs. First, we defined an edit distance between pangenome graphs as the sum of segmentation differences across common genomes that are embedded in both graphs. Second, this method makes it possible to pinpoint where those differences are located along each genome. We believe that understanding how graphs differ is a key step to improve graph construction tools as well as downstream variant analysis.

Starting and ending positions of variants correspond to breakpoints, and the genome segmentation which result from the alignment determine the graph structure. In this work, we showed that regions that are difficult to align lead to differences in graph topology, which in turn lead to differences in detected variant sets. The density of edits along a genome can be interpreted as a confidence indicator for variants called against this genome, though our tool cannot determine which representation is optimal. This highlights a significant challenge in pangenomics: defining objective functions for graph construction and establishing methods to effectively represent complex variation sites within the graph.

This work emphasizes the importance of the construction method choice. In Minigraph-Cactus, the choice of the reference genome is crucial, as it forms the backbone of the entire graph. Opting for PGGB instead of Minigraph-Cactus also influences the resulting graph. Swapping the reference genome in Minigraph-Cactus or using PGGB results in a similar order of magnitude for the number of edits between the graphs, which also translates into differences in numbers of graph-private variants. As a community, we lack a clear understanding of the best practices for pangenome graph construction, and there is a dire need for methods to assess the quality of the graph, or in other words how accurately the graph represents the relationships between individual genomes.

This distance definition does not capture the full picture of the differences between two graphs. Two graphs may share identical genome segmentations but have different topologies. For example, a label shared by multiple haplotypes (or multiple regions of a given haplotype) could be represented in one or several nodes with different connections of these nodes to the rest of the graph without modifying the genome segmentations. These differences can be connectivity differences or differently shared subpaths or even creation of cycles. While our distance is a good proxy on how variants are called, other distances are also possible.

When measured globally over the whole graph, we chose to sum the segmentation distances over all the genomes. This implies that an edit involving a node shared by several genomes is accounted for each time we cross it in the different genomes. The over-weighting of some edits compared to others could be discussed. We argue that differences on nodes traversed by many genomes imply differences in variant representations impacting more genomes, therefore having a greater influence on downstream analyses and justifying their greater weight in the global distance measure. Finally, we can note that this weighting of node edits disappears in the analyses that project the number of edits along one of the genomes.

One of the questions that arises is how graph normalization would impact this distance. Some edit peaks are related to tandem repeats and low-complexity regions. While those factors do not explain all the differences, a significant part of our edits are confined to the tips of the largest nodes or to small nodes, which can be thought as alignment choices that might be resolved through a normalization process. In the context of the representation of the majority of indels, left-normalization is widely used, and may be a satisfactory way to mitigate this issue. To our knowledge, no such tool or algorithm currently exists for graphs, but standardizing variation graphs could be a good way to eliminate biases and ensure consistent results from any graph built from the same data (Audano and Beck 2024).

Our distance metric provides insights into the genome breakdown within the graph, and we extended our analysis to propose hypotheses regarding these differences. Although we explored features that could explain graph differences, we do not have precise answers on good practices to build a pangenome graph. Building pangenome graphs remains a complex task, that requires a careful choice of the genomes that will make the backbone of the graph. It also requires a critical assessment of the pangenome graph builders, so that variants are accurately modeled in the graph construction process. With different existing building methods, we hope that our work can further help identify low-confidence variants and facilitate the investigation of variation representation in pangenome graphs, and open discussions on pangenome graph benchmarking and quality assessment.

## Supplementary Material

btaf291_Supplementary_Data

## Data Availability

*The data underlying this article are available in* Zenodo, at https://doi.org/10.5281/zenodo.10932489
