# Supplementary material for: Pairwise graph edit distance characterizes the impact of the construction method on pangenome graphs
Source: Bioinformatics. 2025 May 9;41(6):btaf291. doi: 10.1093/bioinformatics/btaf291 (PMC12187062; doi:10.1093/bioinformatics/btaf291)
Supplement: btaf291_Supplementary_Data [file btaf291_supplementary_data.pdf]

Pairwise graph edit distance characterizes the impact of the construction method on pangenome graphs

Supplementary Materials

Siegfried Dubois[1,2], Matthias Zytnicki[3], Claire Lemaitre[2], and Thomas Faraut[1]

[1] *GenPhySE, Université de Toulouse, INRAE, ENVT, 31320 Castanet-Tolosan, France*
[2] *Univ Rennes, CNRS, Inria, IRISA - UMR 6074, F-35000 Rennes, France*
[3] *Unité de Mathématiques et Informatique Appliquées, INRAE, Chemin de Borde Rouge, F-31320, France*

Supplementary Table 1

Table S1: Graphs from the HPRC year 1 created with MC and PGGB did not use the same scaffold attribution method, resulting in graphs with a majority of sequences shared in-between them, but also with some private sequences. It is important to underline that the choices were not made by the pangenome builders but during the data curation. As our method focuses on segmentation distances, we could not compare sequence paths that were present in one graph and not in another. It is hard to tell to which extent the presence and absence of those scaffolds in the graphs ended up changing the topology of the graph. However we can still use the yeast graphs as a ground standard as they both use strictly the same input sequences, offering a strict comparison between the two graph builders.

	Yeast, chr1	Human, chr21	Human, chr1
Total MC size	3,172,121	3,770,658,781	21,735,427,417
Total PGGB size	3,172,121	4,012,006,987	22,552,620,745
Shared size	3,172,121	3,648,504,353	21,734,915,199
Private MC size	0	122,154,428	512,218
Private PGGB size	0	363,502,634	817,705,546

Supplementary Figure 1

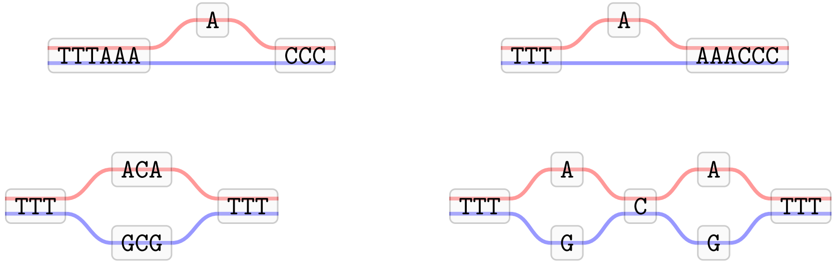

Figure S1: Simple examples of graph differences involving differences in genome segmentations and in the representation of genomic variants.
The two graphs on the upper part of the figure represent the same genomes. The red genome has an additional `A` (in a stretch of three other `A`s) compared to the blue genome. The position in the graph of this extra `A` within the `A` stretch is arbitrary, and mostly a choice of the method. Choosing one of the candidates `A`s changes the breakpoint positions on the genomes. The variant sequence is the same (`A`) but it has different positions (position 6 for the left graph and position 3 for the right graph).
The two graphs on the bottom part also represent the same genomes. On the right graph, the two middle nodes of the left graph are split into three nodes each, which adds 4 breakpoints to each genome. The left graph represents a single variant (`ACA / GCG` at position 3) where the right graph represents two variants (`A / G` at position 3 and `A / G` at position 5).

Supplementary Figure 2

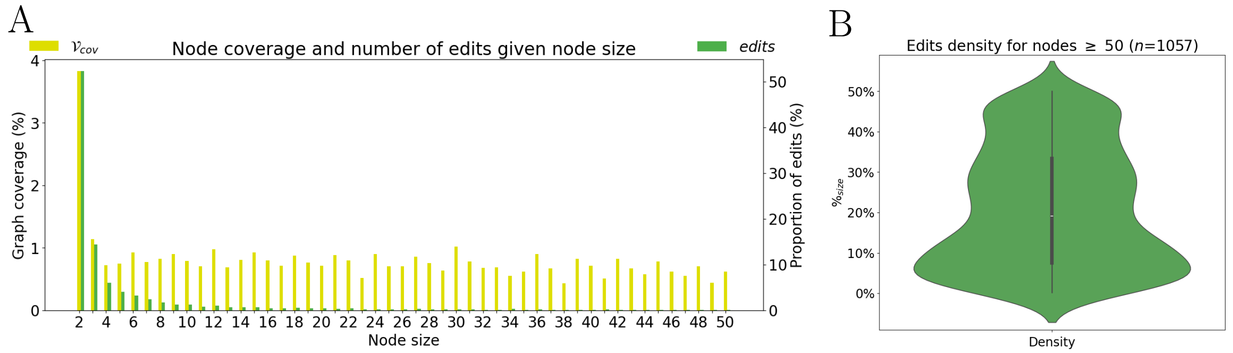

Figure S2: Distribution of edits over the nodes of the graph for the comparison of MC and PGG yeast graphs. A) For every node size n between 2 and 50 bp, we represent in yellow the percentage of the graph covered by nodes of size n (left axis), and in green the proportion of edits localized on nodes of size n. For instance, the nodes of size 2 cover 3.82% of the total graph size but contain 52.23% of the edits. Nodes of size <50 bp covers 39.51% of the total graph size but contain 97.35% of the edits. B) Edit position on nodes, expressed as a percentage of size for long nodes (≥50 bp). We can observe that more edits are located near the borders of the nodes than in the middle. Chi-Square Goodness-of-Fit test ($\chi^2 = 110.31$, $p < 0.0001$) rejects uniformity, confirming a non-uniform position distribution.

Supplementary Figure 3

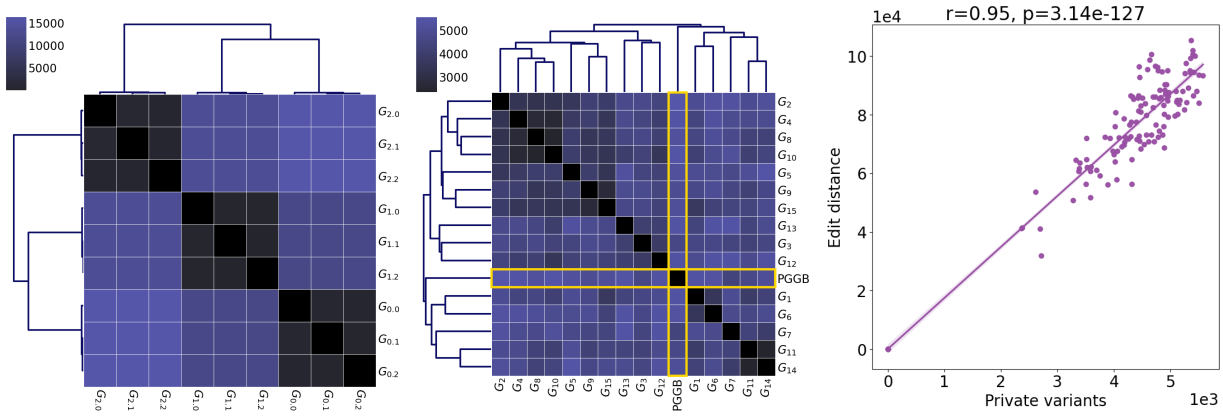

Figure S3: Hierarchical agglomerative clustering of graph-private variants between yeast graphs. The compared graphs are the same as the ones from Fig. 1 of the main manuscript. A lower count means that fewer variants are specific to one of the graphs. All variants have been called against the same reference genome. The clustering of graphs based on their number of graph-private variants is similar to the clustering based on our edit distance.

Supplementary Figure 4

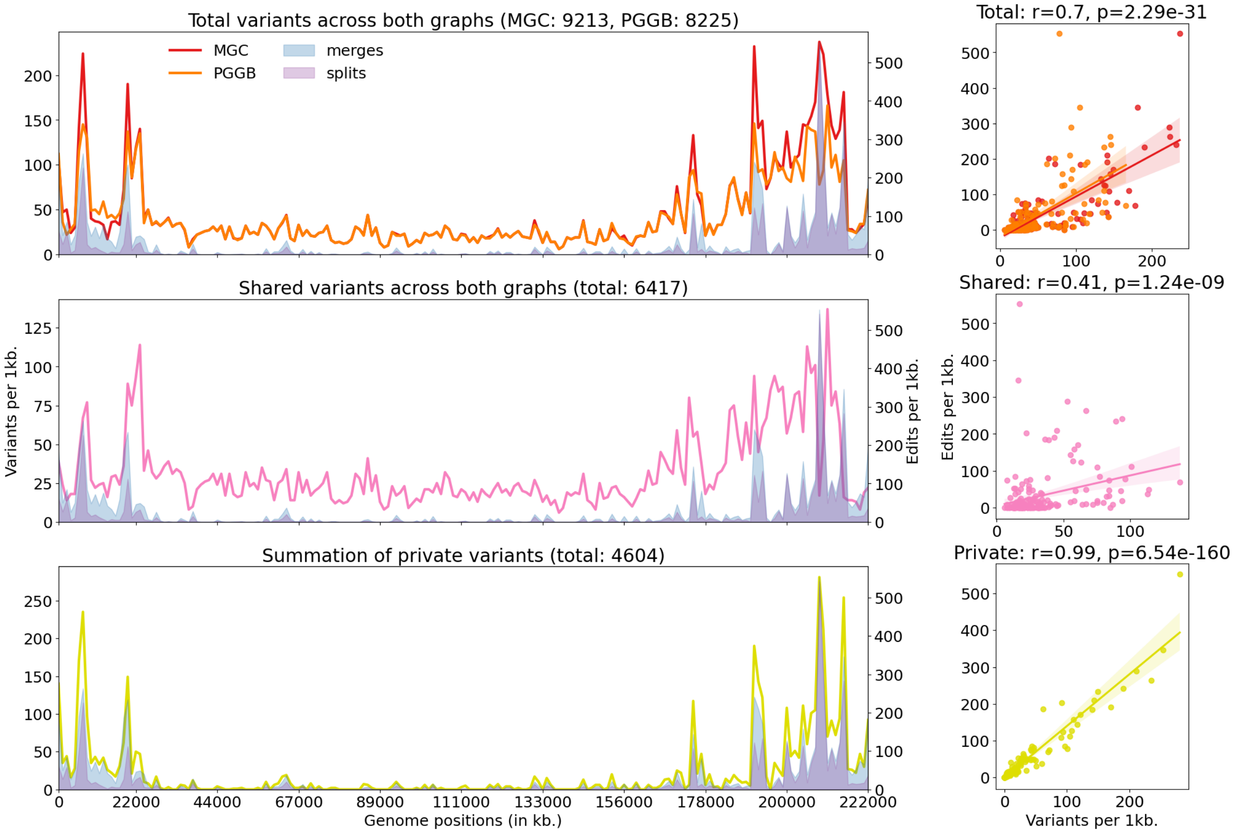

Figure S4: Distributions along the reference genome (CASBJU01) of the number of edits and number of variants, between yeast graphs built with MC and PGGB in 1 kb windows. Variants were computed for both graphs against this same reference sequence. Total variants counts are displayed in the top figures, while the middle and bottom figures consider subsets of the variant sets: variants shared by both graphs (i.e. identical representations in both VCF files) in the middle figure, and variants specific to one or the other graph, graph-private variants in the bottom figures. Figures on the right show scatter plots of these numbers computed in 1kb windows with r being the Spearman correlation coefficient.

Supplementary Figure 5

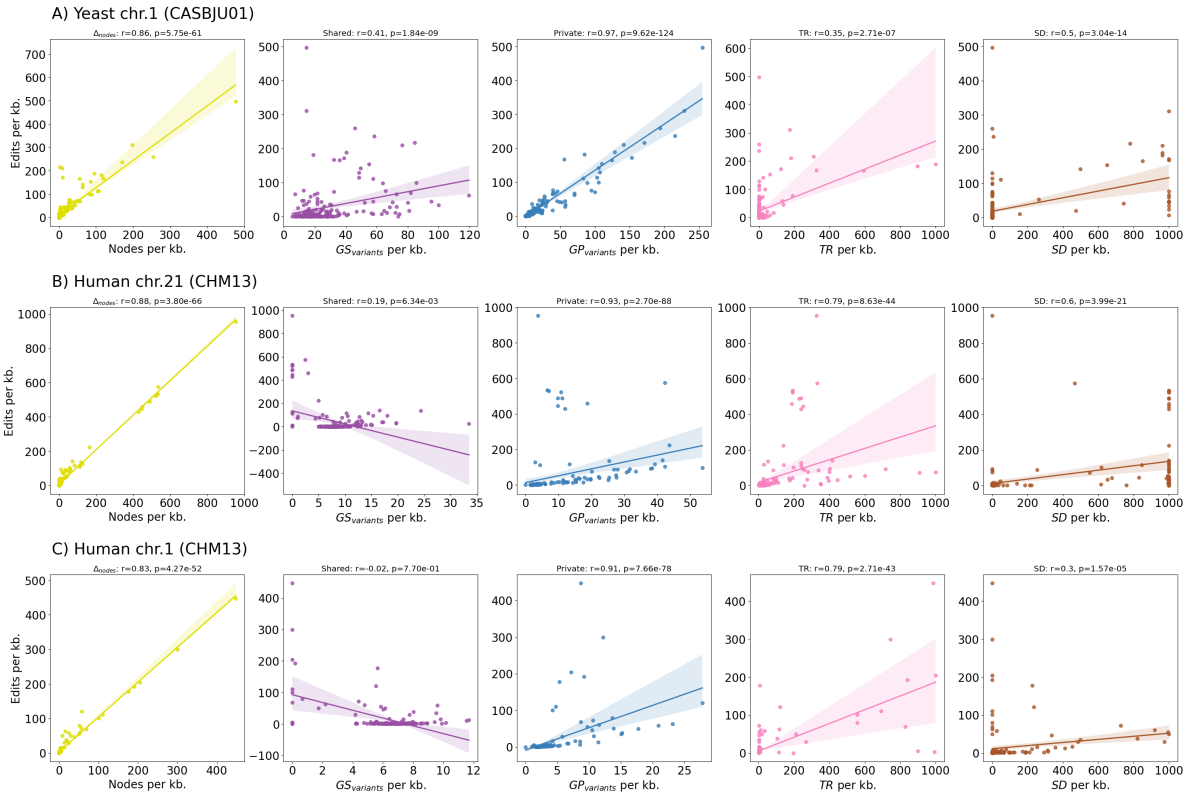

Figure S5: Scatter plots depicting the relationships between the number of edits per kilobase and five graph or genomic features computed along the reference genome: node count, number of graph-shared variants and graph-private variants, number of bases covered by tandem repeats and segmental duplications, all expressed per kilobase (left to right). The plots for the three chromosome graphs are shown in rows (yeast chromosome 1, human chromosomes 21 and 1). Spearman correlation coefficients and their associated p-values are reported, testing the null hypothesis that the two densities have no ordinal correlation.

Supplementary Text 1

Here, we prove that our metric is indeed a distance. The following properties are given at the path level (segmentation distance). As the sum of distances are a distance, if d_s is a distance then d is a distance too:

- *Positive*: our metric is a summation of the number of elements of two sets, $\mathcal{M}$ and $\mathcal{S}$. Those counts can be positive or zero (meaning segmentation are identical), and from Algorithm 1, having zero merges and zero splits means that we have the same segmentation on both paths, thus validating $d_s(\mathcal{P}_i^a, \mathcal{P}_i^b) = 0 \Leftrightarrow \mathcal{B}^a = \mathcal{B}^b$
- *Symmetry*: merge and split are reciprocal operations. From the definition of the path segmentation distance, if we find the minimal couple of sets $\langle \mathcal{M}^a, \mathcal{S}^a \rangle$, we can define the reciprocal couple $\langle \mathcal{M}^b, \mathcal{S}^b \rangle$ where $\mathcal{M}^b = \mathcal{S}^a$ and $\mathcal{S}^b = \mathcal{M}^a$, thus granting symmetry as we keep the same number of total operations.
- *Triangle inequality*: let $\mathcal{P}_\alpha^a$, $\mathcal{P}_\alpha^b$ and $\mathcal{P}_\alpha^c$ be three paths in three distinct graphs representing a same genome Γ_α. We need to show that $d_s(\mathcal{P}_\alpha^a, \mathcal{P}_\alpha^c) \leq d_s(\mathcal{P}_\alpha^a, \mathcal{P}_\alpha^b) + d_s(\mathcal{P}_\alpha^b, \mathcal{P}_\alpha^c)$. It exists for each segmentation a set of breakpoints $\mathcal{B}$. We can transform our equation into $\mathcal{B}_\alpha^a \ominus \mathcal{B}_\alpha^c \leq \mathcal{B}_\alpha^a \ominus \mathcal{B}_\alpha^b + \mathcal{B}_\alpha^b \ominus \mathcal{B}_\alpha^c$, which is true from the definition of the symmetric difference.
